# Zinc and nitrogen mediate the regulation of growth, leading to the upregulation of antioxidant aptitude, physio-biochemical traits, and yield in wheat plants

**DOI:** 10.1038/s41598-024-63423-y

**Published:** 2024-06-05

**Authors:** Nimra Shehzadi, Athar Mahmood, Muhammad Kaleem, Muhammad Shahbaz Chishti, Humaira Bashir, Abeer Hashem, Elsayed Fathi Abd-Allah, Hina Shahid, Atiqa Ishtiaq

**Affiliations:** 1 Department of Botany, University of Agriculture, Faisalabad, 38040 Pakistan; 2 College of Life Sciences, Northeast Forestry University, Harbin, 150040 China; 3 Government Graduate College for Women Wahdat Colony, Lahore, Pakistan; 4 Institute of Botany, University of the Punjab, Lahore, Pakistan; 5 Botany and Microbiology Department, College of Science, King Saud University, P.O. Box. 2460, 11451 Riyadh, Saudi Arabia; 6 Plant Production Department, College of Food and Agricultural Sciences, King Saud University, P.O. Box. 2460, 11451 Riyadh, Saudi Arabia; 7 Department of Botany, Government College University, Faisalabad, Pakistan; 8 Department of Agronomy, University of Agriculture, Faisalabad, 38040 Pakistan

**Keywords:** Antioxidants, Grain quality, Growth, Lipid peroxidation, Nutrients, Photosynthetic pigments, Plant sciences, Environmental sciences

## Abstract

An ample amount of water and soil nutrients is required for economic wheat production to meet the current food demands. Nitrogen (N) and zinc (Zn) fertigation in soils can produce a substantial wheat yield for a rapidly increasing population and bring a limelight to researchers. The present study was designed to ascertain N and Zn’s synergistic role in wheat growth, yield, and physio-biochemical traits. A pot experiment was laid out under a complete randomized design with four N levels (N1-0, N2-60, N3- 120, and N4-180 kg ha^−1^), Zn (T1-0, T2-5, T3-10, and T4-15 kg ha^−1^) with four replications. After the emergence of the plants, N and Zn fertigation was applied in the soil. The growth traits were considerably increased by combined applications as compared to the sole applications of the N and Zn. The photosynthetic pigments were found maximum due to combined applications of N and Zn, which were positively associated with biomass, growth, yield, and wheat grain quality. The combined application also substantially enhances the antioxidant enzyme activities to scavenge the ROS as H_2_O_2_ and reduce lipid peroxidation to protect the permeability of the biologic membranes. The combined higher applications of N and Zn were more responsive to ionic balance in a shoot by maintaining the Na^+^ for osmotic adjustments, accumulating more Ca^2+^ for cellular signaling; but, combined applications resulted in K^+^ reduction. Our present results suggest that appropriate sole or combined applications of N and Zn improve wheat's growth, yield, and antioxidant mechanisms. Previous studies lack sufficient information on N and Zn combined fertigation. We intend to investigate both the sole and combined roles of N and Zn to exploit their potential synergistic effects on wheat.

## Introduction

The deficiency of dietary micro and macronutrients is spreading rapidly and poses a severe threat to more than 2 billion people around the globe^1,2^. Nowadays, the increase in productivity of the crops is a notable concern for plant researchers rather than improving the taste and quality of the staple crops^3,4^. Due to the soil's low nutrients, malnutrition onset is common in wheat and other staple crops. This nutritional deficiency is regarded as a more challenging task for scientists^5,6^. Among micronutrients, Zn deficiency causes digestive problems^7^, reduced reproductive health, stunted growth, and a mutation in DNA structure, which leads to malignancy, hormonal disturbances, diabetes mellitus, and a high risk of cardiovascular problems in humans^8^. Among macronutrients, a low supply of N to humans results in disruptions in protein synthesis and negative impacts on brain functioning and the immune system^9^. In many developing countries, the primary concern is food and nutritional security and providing ample food to people, but these goals are still unattainable^10^.

Wheat (*Triticum aestivum* L.) stands out as a valuable and indispensable food crop for modern times^11^. Wheat, as a cereal crop, embraces global importance and is used as a staple crop by 1.2 billion people throughout the world^12^. Annually, wheat is cultivated at 214.7 million hectares (M hac) and is considered the second major food crop after maize, producing 749 million tons^13^. In Pakistan, wheat is cultivated at 8.79 M hac of agricultural land, and the production surplus is the country's need, with a production of 25.076^14^. In rural Pakistan, people intake 70% of calories from wheat and use it as an important dietary food. Also, wheat is used as a crucial dish^15^. Wheat controls nutritive risks in developing countries where food insecurities are common and feed people^16^. But, in most developed countries, a lack of entire grains is the main nourishing risk factor for death^17^. The world population is escalating rapidly and will increase by 40% by 2050. To meet food security, the yield can increase with population increase. Wheat is an important source of carbs and gluten, an important element for making dairy and baking products^18^. Also, in comparison, it surpasses all agronomic crops by agronomic yield. Even wheat straw is used as feed for livestock^19^. Excessive and monotonous use of wheat by humans in routine life causes malnutrition and wastage^20^. To attain optimum yield and fulfill food demands, a sufficient amount of organic and inorganic fertilizers with Zn and N can be added to soils with low-nutrient profiles^21^. Moreover, in addition to growth nutrients, the use of correct fertilizer has become a good source for sustaining agronomic yield^22^.

The nutrient deficit in soils is increasing day by day due to poor agriculture practices, poor irrigation, repetitive cropping, frequent growing crops for high yield, and intensive use of P, K, and N fertilizers^23^. Studies suggest approximately 50% of wheat-growing areas worldwide are experiencing Zn deficiency^24^. In Pakistan, the fertile soil has raised pH and electrical conductivity, excessive accumulation of calcium carbonate, more cations, more bicarbonates, and scarcity of N and Zn^25^. Zinc is an important structural constituent of the vital enzyme important for protein synthesis and hormone regulations, which plants use to promote plant growth, pollination, nucleic acid, carbohydrate metabolism, and starch synthesis in grain^26^. As a micronutrient, zinc enhances the activities of aldolases and carbonic anhydrases to regulate carbon metabolism^27^.

Additionally, Zn helps to regulate photosynthesis by increasing photosynthetic pigments soluble sugars and regulating seed and pollen grain formation^28^. Exogenously applied Zn can regulate gene expression in a stomatal aperture to withstand oxidative damage due to nutritional imbalance^29^. Plants grown on low-profile soils show evident negative symptoms, including reduced leaf area, inhibited growth, leaf chlorosis, and spikelet sterility^30^. But, high amounts of zinc can cause toxicity, which leads to leaf cracking, abnormal shoot elongation, and negatively influences plant biomass and yield^31^.

Nitrogen (N) is a decisive inorganic element that increases plant growth, leaf area, radiation interception by leaves, leaf emergence, photosynthetic capacity, and root vigour^32^. Nitrogen is mainly taken up by the root epidermal extensions and transported via the apoplast pathway toward the xylem fibres with the help of carrier-mediated channels such as nitrate transporters and ammonium transporters through facilitated diffusion^33^. The N in the form of nitrate is assimilated more in vegetative leaves than in the roots^34^. To produce ample yield in wheat, an appropriate amount of N fertilizer can be used as a key element^35^. Therefore, N fertigation to nutrient deficit soils can deeply enhance leaf biomass at the anthesis and booting stages to increase leaf area index, root, and shoot vigour. The balanced supply of N enhances photosynthesis; better photosynthesis responded positively to increased spikes, grain numbers and grain quality^36^. Nitrogen availability at earlier stages prompted vegetative growth and yields, as well as increased protein and starch content in grains. Conversely, N applications intercept the negative effects on grain yield and quality at post/late growth stages^37^.

In the current era, different grain bio-fortification methods are used. In contrast, agronomic bio-fortification in cereal crops is considered a rapid and economical tool due to its low cost and sustainable strategy to improve wheat productivity, growth, and nutrient profiling. To meet wheat demands, N fertilizers are a critical tool for attaining the required productivity. The above-mentioned ideas suggest that wheat grown in soils with poor nutrient levels leads to malnutrition and seriously threatens humans and plants. Hence, it is a concern of the current era to enhance the nutritional contents of wheat and soils through numerous agronomic practices. A promising strategy that could help cereal producers improve nitrogen use efficiency is gene editing or molecular tools^38^. In previous findings, agricultural scientists primarily focused on the implications of single-element fertilizers on cereals, yet limited information is available on the combined fertigation of N and Zn. Present notations suggest we explore the combined and sole applications of the N and Zn on yield, growth, and physio-biochemical attributes. Moreover, we intend to determine the impact of N and Zn applications on shoot ions, cellular antioxidants, and organic osmolytes. We hypothesized that N and Zn fertigation might help improve wheat yield and grain quality through physiological and biochemical processes.

## Materials and methods

### Experimental conditions, establishment, and layout

The seeds of one wheat genotype Akbar-19 seeds were procured from Ayub Agricultural Research Institute, Faisalabad-Pakistan. Pot experimentation was carried out in the research area of University of Agriculture community college, Postgraduate Agricultural Research Station (PARS) (coordinates: 31.3848^o^N, 72.996^o^E, altitude 184.4 m a.s.1). Seeds of wheat were engrossed in 30% hydrogen peroxide (H_2_O_2_) for 1 h for microbial disinfection, thoroughly washed with distilled water (H_2_O) for 2 h, dried and stowed until sowing. Ten uniformly selected seeds were sown into each plastic pot (630 cm^3^) for growth and filled with soil (3 kg/pot). The soil in plastic pots has a sandy loam texture (Electrical conductivity/ECe 1.2 dS m^−1^, pH 8.22, Na^+^ 39.95 mg kg^−1^, Cl^−^ 9.98 mg kg^−1^, Mg^2+^ 250 mg kg^−1^, organic matter-OM 0.87%, Zn 9.88 mg kg^─1^, N 599.3 mg kg^−1^, P 7.5 mg kg^−1^ and K 123 mg kg^−1^. After seedling emergence, plants were thinned to six for better growth to prevent nutrient deficiency until analysis and harvesting. The experimental units were arranged as completely randomized design with four replications for each treatment with a day/night temperature of 27 °C/21 °C and relative humidity of 55–65%. After sowing, half-strength Hoagland’s nutrient solution (4.66 mM Ca (NO_3_), 2⋅4 H_2_O, 1.41 mM KH_2_PO_4_, 4.98 mM KNO_3_, 1.99 mM MgSO_4_⋅7H_2_O, 0.10 mM FeSO_4_⋅7H_2_O, 0.10 mM ethylene diamine tetra acetic acid (EDTA), 46.26 μM H_3_BO_4_, 9.10 μM MnCl_2_⋅4H_2_O, 0.77 μM ZnCl2, 0.41 μM CuCl_2_⋅2H_2_O, and 0.13 μM Na_2_MoO_4_⋅2H_2_O) was uniformly added to all plastic pots for an interlude of 2 days until the development of seedlings took place. The field capacity was maintained throughout the work to prevent water deficit conditions. After each seven-day gap, the pots were reshuffled throughout the experimentation to avoid border and pseudo-replication effects.

### Treatments

After seed emergence, seeds were exogenously treated with Zn and N treatments. Different levels of Zn and N were applied to each pot. Each N treatment has four regimes: N1-0, N2-60, N3-120, and N4-180 kg ha^−1^. Each Zn treatment has four levels: T1-0, T2-5, T3-10, and T4-15 kg ha^−1^.

### Plant sampling

Plant sampling was done at the time of maturity until reaching growth stage 75 on the Zadok’s scale (after 110 days of seed sowing) by following the method outlined by Zadoks et al.^39^. Fully matured flag leaves from each treated pot were taken and immediately stored at − 70 °C to determine the physiological and biochemical parameters, reactive oxygen species (ROS), and antioxidants assay. Harvesting was done after the maturity and growth and yield components were recorded. For this purpose, the floral parts, such as the leaf, shoot, and leaf, were washed with distilled water to remove the soil, and growth traits were recorded.

### Plant analysis and measurements

#### Measurement of growth traits

After harvesting, five plants from each treatment were used to determine the root and shoot fresh and dry biomass by using a digital weighing balance (± 0.01gm), while dry weight was taken after oven drying the samples for 72 h at by scale (± 0.001 gm) at 70 °C. After harvest, each plant's roots and shoot were separated from the plant and delicately washed by running tap water. A manual measuring scale recorded the root length and shoot length. Plant height was recorded from root to leaves with the help of a measuring scale. The leaves number was visually counted from each experimental pot under different treatments. The leaf area (LA) was measured using the formula L × W × 0.75. The leaf area ratio (LAR) was measured by ascertaining the ratio between the leaf area (LA) and shoot dry weight (SDW) of the plants by using the following equation;

1 $${\text{LAR }} = {\text{ LA}}/{\text{ SDW}}$$

#### Yield components

 The number of fertile tillers (m^-2^) undertaken from each treatment. The length of spikelets was measured with measuring tape, and spike length (SL) was measured precisely. Manual calculations were used to find the number of spikes and spikelets per spike. After threshing, the grain's numbers were counted and averaged per plant.

#### Leaf photosynthetic pigments

Plant fresh leaf (0.25gm) were homogenized in an 80% acetone solution at 0-4o C to ascertain the photosynthetic pigments as chlorophyll a, b, and total chlorophyll (*T.Chl*) contents by following the Arnon, 1949^40^ method and carotenoids were ascertained by Goodwin^41^. The extracted material was then filtered by using filter paper. The filtrate was centrifuged at 10,000 × at 4 °C for 5 min. Pigment absorption was noted at 480, 645, and 663 nm with the help of an ultraviolet–visible spectrophotometer (Hitachi-220, Japan).

#### Cellular antioxidants

To prepare enzyme extract, wheat leaves (0.5gm) were ground in 1% polyvinyl pyrrolidine, 1 mM ethylene diamine tetra acetic acid (EDTA), 0.05 M sodium phosphate buffer having a volume of 5 mL in a pestle mortar. The supernatant was collected after centrifugation at 20,000 rpm and 4 °C for 20 min to analyze antioxidant enzymes. The protein contents were estimated using method^42^, and bovine serum albumin was used to calculate the enzyme-specific activity.

#### Super oxide dismutase

The activity of superoxide dismutase (SOD) was recorded based on the inhibition level of nitro blue tetrazolium (NBT) reduction^43^. The 10 µL of extracted enzyme solution was added in 3 mL of reactants (200 mM methionine, 100 mM (Na_3_PO_4_) phosphate buffer, 1.5 Na_2_CO_3_, 3 mM ethylene diamine tetra acetic acid (EDTA), 2.25 NBT, 0.06 mM concentration of riboflavin, and distilled water. The same set of test tubes was prepared as a control. Fluorescent bulbs irradiated both reactant and control test tubes for 15 min. After that, the test tubes were darkened with a black cloth for reaction termination purposes. A blank was prepared following the above method without irradiation. The levels of optical density (OD) were determined at 560 nm. One unit of SOD accounted for the quantity of enzyme inhibiting 50% of NBT photoreduction.

#### Peroxidase

The activity of peroxidase (POD) was determined in the presence of H_2_O_2_ by following the method of^44^. A reactant solution was prepared in glass test tubes containing 3 mL of phosphate buffer, 10 mM H_2_O_2_, and 20 mL of guaiacol. The reaction was started after adding the enzyme extract, and specific absorption was recorded spectrophotometrically at 460 nm.

## Oxidative stress markers

### Hydrogen peroxide

The quantification of H_2_O_2_ was based on ferrous ions oxidation induced by POD concentration. These ferrous ions react with xylenol in reactants^45^. The leaf material (0.5 gm) was grounded in sodium phosphate buffer, and homogenized was centrifuged at 10,000 × for 15 min. The supernatant was collected and reacted with the assay reagent. After 45 min, absorbance was recorded at 560 nm.

### Lipid peroxidation/MDA

Lipids peroxidation (LPX) was measured using an amount of malondialdehyde (MDA)^46^. Thiobarbituric acid (TBA) and MDA were used to determine LPX concentration. The 250 mg leaf sample was homogenized in 5 mL of 0.1% (w/v) trichloroacetic acid (TCA) and centrifuged at 15,000 × g. The 1 mL aliquot was mixed with 2 mL of 0.5% TBA and 20% TCA. Reactants were placed in test tubes then incubated at 85 °C for 10 min, and the reaction was stopped in an icebox. The optical density was determined at 532 and 600 nm by spectrophotometer (Hitachi U2910, Tokyo, Japan). The optical density (532 -600 nm) was used to determine the final values. The 155 mM cm^-1^ as an extinction coefficient was used to count LPX.

### Shoot ionic contents

Ionic contents in the shoot were recorded using the Wolf method^47^. The plant sample (0.25 g) dried and was added to digestion tubes with 5 mL of concentrated H_2_SO_4_ and then incubated overnight. After incubation, 500 ml of 30% H_2_O_2_ (V/V) was added. The test tubes were placed on a digestion plate, and the temperature was gradually raised till the formation of fumes at 350 °C. When the mixture cooled, then 30% H_2_O_2_ was added. The process was repeated until a clear solution was formed. Deionized water was added to digested material to dilute it up to 50 ml. using a flame photometer, shoot K^+^ Na^+^ and Ca^2+^ were determined (Portable flame photometer-PFP7, Jenway, and Staffordshire, United Kingdom).

### Statistical analysis

The data visualization and statistical analysis were performed using the R statistical software (R Core Team, 2022) using the R integrated development environment R Studio (R Studio Team, 2022). Curated data were subjected to a two-way analysis of variance (ANOVA) to evaluate the interaction between N and Zn treatment at a significance level (*p* ≤ *0.05)*. The treatment means standard errors (SE) and level of significance (ANOVA) were compared by Tukey’s honestly significant difference by using the R-integrated “Agricolae” package. The principle component analysis was done to evaluate the relationship between a variable of different traits and the possible impact of treatment with the help of R code “FactoMineR and ggplot2. The Clustered heatmap was constructed by R integrated code “pheatmap, while the correlation matrix was drawn by “GGally.

### Ethics approval

The pot experiment and plant material collection were conducted per relevant institutional and national guidelines and legislation.

## Results

### Growth attributes

The exogenous application of nitrogen (N) and zinc (T) considerably increases the growth traits of wheat. The sole applications of Zn notably increase wheat's shoot fresh weight (SFW); a more pronounced increase in FW occurred in response to the T4 level. The collective applications of Zn and N positively influence SFW, and the maximum mean value for this trait was evaluated for N4:T4. We observed a similar response for the shoot dry weight by applying Zn and N. The root fresh and dry weights were substantially increased as a result of both nutrient applications. The maximum mean values were found in response to T4 and N4 by showing a linear increase as application levels were increased. The root length (RL) and shoot length (SL) were considerably increased by an increase in the levels of Zn, the highest mean value drawn by T4. The combined application of Zn and N notably enhanced the RL and SL in response to higher regimes. The combined application of both nutrients causes a considerable increase in plant height (PH) and leaves per plant (NLPP). The maximum value for NLPP and plant height (PH) was secured by T4:N4 (Table 1).

**Table 1:** Growth traits of wheat treated with different levels of Zn and N. Means ± SE provided with error bars; different letter indicates significance (*p* ≤ 0.05) between N and Zn treatments.

N_2_ Levels (Kg/hac)	T1	T2	T3	T4
SFW (g plant^−1^)				
N1	2.65 ± 0.27^Bc^	2.68 ± 0.47^Cc^	2.86 ± 0.52^Bb^	2.89 ± 0.15^Ba^
N2	2.28 ± 0.24^Cc^	2.76 ± 0.42^Ca^	2.65 ± 0.26^Cb^	2.75 ± 0.53^Ba^
N3	2.13 ± 0.64^Cc^	2.80 ± 0.77^Ba^	2.76 ± 0.16^Bb^	2.74 ± 0.03^Ca^
N4	3.17 ± 0.63^Aa^	3.18 ± 0.38^Aa^	2.93 ± 0.42^Ab^	3.15 ± 0.01^Aa^
SDW (g plant^−1^)				
N1	0.61 ± 0.13^Cc^	0.77 ± 0.05^Bb^	0.73 ± 0.13^Cb^	0.85 ± 0.13^Ba^
N2	0.63 ± 0.11^Cc^	0.70 ± 0.11^Bb^	0.72 ± 0.15^Cb^	0.90 ± 0.09^Aa^
N3	0.70 ± 0.10^Bc^	0.84 ± 0.12^Ab^	0.83 ± 0.07^Bb^	0.93 ± 0.09^Aa^
N4	0.80 ± 0.13^Ab^	0.92 ± 0.05^Aa^	0.94 ± 0.05^Aa^	0.95 ± 0.01^Aa^
RFW (g plant^−1^)				
N1	0.15 ± 0.04^Cc^	0.23 ± 0.02^Cb^	0.27 ± 0.02^Bb^	0.36 ± 0.02^Ba^
N2	0.18 ± 0.01^Cc^	0.25 ± 0.04^Cb^	0.28 ± 0.03^Bb^	0.39 ± 0.01^Ba^
N3	0.23 ± 0.04^Bc^	0.28 ± 0.03^Bb^	0.32 ± 0.05^Bb^	0.44 ± 0.04^Ca^
N4	0.33 ± 0.01^Ac^	0.34 ± 0.01^Ac^	0.37 ± 0.01^Ab^	0.56 ± 0.01^Aa^
RDW (g plant^−1^)				
N1	0.02 ± 0.04^Cb^	0.05 ± 0.03^Ca^	0.05 ± 0.00^Ca^	0.05 ± 0.01^Ca^
N2	0.05 ± 0.00^Bb^	0.06 ± 0.01^Cb^	0.07 ± 0.01^Bb^	0.08 ± 0.01^Ba^
N3	0.07 ± 0.01^Bb^	0.09 ± 0.04^Ba^	0.07 ± 0.01^Bb^	0.09 ± 0.01^Ba^
N4	0.09 ± 0.03^Ac^	0.11 ± 0.04^Ab^	0.10 ± 0.01^Ab^	0.12 ± 0.03^Aa^
RL (cm)				
N1	4.17 ± 0.73^Bd^	5.17 ± 0.73^Cc^	8.80 ± 1.25^Cb^	9.30 ± 1.17^Ca^
N2	10.17 ± 0.93^Ab^	9.13 ± 1.74^Bb^	12.40 ± 0.32^Ba^	12.07 ± 1.23^Ba^
N3	10.00 ± 2.75^Ab^	10.00 ± 1.26^Ab^	13.77 ± 1.68^Ba^	14.50 ± 1.80^Ba^
N4	11.27 ± 2.71^Ab^	12.27 ± 1.23^Ab^	16.27 ± 1.47^Aa^	18.83 ± 1.48^Aa^
SL (cm)				
N1	13.00 ± 1.53^Cd^	14.10 ± 1.25^Cc^	24.83 ± 5.36^Bb^	32.43 ± 2.83^Ba^
N2	19.00 ± 3.18^Bc^	19.60 ± 1.71^Bc^	28.87 ± 10.18^Ab^	33.47 ± 5.18^Ba^
N3	22.50 ± 2.02^Ac^	22.13 ± 4.20^Bc^	30.87 ± 0.38^Ab^	36.00 ± 3.36^Aa^
N4	24.33 ± 6.96^Ac^	26.23 ± 0.67^Ac^	33.30 ± 1.75^Ab^	37.30 ± 3.47^Aa^
PH (cm)				
N1	32.97 ± 0.75^Cc^	43.03 ± 0.62^Cb^	52.60 ± 3.50^Ca^	54.43 ± 2.28^Ca^
N2	36.73 ± 7.47^Cc^	50.27 ± 0.92^Bb^	56.87 ± 0.98^Bb^	60.00 ± 1.00^Ba^
N3	40.83 ± 7.57^Bc^	58.57 ± 1.98^Ab^	58.67 ± 3.83^Bb^	63.83 ± 0.42^Ba^
N4	48.07 ± 2.21^Ac^	60.67 ± 0.94^Ab^	66.23 ± 6.42^Aa^	69.03 ± 0.03^Aa^
NLPP				
N1	2.67 ± 0.33^Bc^	3.33 ± 0.33^Cb^	4.67 ± 0.33^Ca^	4.33 ± 0.67^Ca^
N2	3.00 ± 0.58^Bc^	5.33 ± 0.67^Ba^	5.00 ± 0.00^Bb^	5.67 ± 0.33^Ba^
N3	4.33 ± 0.33^Ac^	5.00 ± 0.00^Bb^	5.67 ± 0.33^Ba^	5.00 ± 0.58^Bb^
N4	4.00 ± 0.00^Ac^	6.33 ± 0.33^Ab^	6.00 ± 0.58^Ab^	7.00 ± 0.58^Aa^

Means provided with error bars. The small letter indicates a significant (*p* ≤ 0.05) difference between Zn treatments and capital letters for N_2_.

For an abbreviation description see the list at the start of the manuscript.

The wheat genotype's leaf area increased as the nutrient treatment levels increased. The N4 with T4 responded to the maximum mean values, which showed a substantial increase. The leaf area ratio (LAR) has not substantially changed in response to T1:N1, T2:N2, and T3:N3. But a LAR notable increase in response to combined applications. The sole applications of Zn and N considerably improved the biologic yield of the wheat plants. The number of fertile tillers (NFT) per plant was maximum in response to higher N and Zn treatments. The number of spikelets showed the same increasing trend line pattern to improve the biologic yield of the wheat plant. No major change in mean values was evaluated for spike length (SPL) at low or higher treatments of the Zn and N. The yield parameters DTH (day to heading) and DTM (days to maturity) showed a substantial increase as the level of the treatment was increased (Table 2).

**Table 2:** Growth and yield of wheat treated with different levels of Zn and N. Means ± SE provided with error bars; different letter indicates significance (*p* ≤ 0.05) between N and Zn treatments.

N_2_ Levels (Kg/hac)	T1	T2	T3	T4
LA (Cm^2^)				
N1	15.23 ± 0.54^Bb^	17.01 ± 0.22^Ca^	19.00 ± 1.22^Ca^	19.12 ± 0.89^Ca^
N2	15.45 ± 0.22^Bc^	19.22 ± 0.25^Cb^	23.11 ± 0.99^Ba^	24.22 ± 0.22^Ca^
N3	16.56 ± 0.12^Bd^	22.32 ± 0.77^Bc^	27.21 ± 0.22^Bb^	33.03 ± 0.89^Ba^
N4	30.30 ± 0.58^Ac^	33.21 ± 0.52^Ab^	37.33 ± 0.96^Aa^	43.98 ± 0.98^Aa^
LAR (m^2^)				
N1	25.02 ± 0.23^Bd^	28.20 ± 0.65^Bc^	35.11 ± 0.23^Ab^	55.01 ± 0.22^Ba^
N2	29.25 ± 0.21^Ac^	28.23 ± 0.52^Bc^	34.22 ± 0.52^Ab^	56.63 ± 0.65^Ba^
N3	29.26 ± 0.33^Ac^	29.63 ± 0.33^Bc^	35.78 ± 0.25^Ab^	59.69 ± 0.32^Aa^
N4	30.25 ± 0.63^Ac^	33.52 ± 0.23^Ab^	37.11 ± 0.55^Ab^	62.56 ± 0.65^Aa^
NFT plant^−1^				
N1	6.32 ± 0.21^Bc^	7.11 ± 0.22^Bb^	8.11 ± 0.96^Aa^	8.02 ± 0.55^Ba^
N2	6.63 ± 0.22^Bb^	8.02 ± 0.52^Aa^	8.21 ± 0.65^Aa^	8.01 ± 0.65^Ba^
N3	6.66 ± 0.09^Bb^	8.77 ± 0.52^Aa^	8.22 ± 0.96^Aa^	8.44 ± 0.33^Ba^
N4	7.01 ± 0.03^Ac^	8.56 ± 0.65^Ab^	8.63 ± 0.87^Ab^	9.21 ± 0.51^Aa^
NOS plant^−1^				
N1	11.11 ± 0.21^Bc^	13.01 ± .22^Bb^	15.00 ± 0.54^Bb^	17.52 ± 0.33^Ca^
N2	12.21 ± 0.54^Bc^	13.32 ± 0.65^Bc^	15.01 ± 0.54^Bb^	17.89 ± 0.21^Ca^
N3	16.22 ± 0.21^Ab^	13.11 ± 0.54^Bc^	16.09 ± 0.21^Bb^	19.54 ± 0.28^Ba^
N4	16.320.32^Ac^	15.63 ± 0.54^Ac^	18.11 ± 0.26^Ab^	22.88 ± 0.99^Aa^
SPL (cm)				
N1	7.65 ± 0.11^Bb^	8.56 ± 0.11^Ba^	8.78 ± 0.22^Ba^	8.65 ± 0.52^Ba^
N2	7.96 ± 0.21^Bb^	8.58 ± 0.09^Ba^	8.23 ± 0.21^Ba^	8.32 ± 0.22^Ba^
N3	8.36 ± 0.12^Ab^	8.96 ± 0.32^Ba^	8.14 ± 0.51^Bb^	8.54 ± 0.65^Ba^
N4	8.98 ± 0.33^Ac^	9.65 ± 0.15^Ab^	9.88 ± 0.85^Aa^	9.90 ± 0.21^Aa^
NG plant^−1^				
N1	232 ± 10.21^Bd^	244 ± 19.66^Dc^	254 ± 32.33^Bb^	265 ± 63.45^Da^
N2	233 ± 9.32^Bc^	265 ± 23.25^Cb^	265 ± 44.25^Bb^	286 ± 62.32^Ba^
N3	236 ± 6.33^Bd^	278 ± 29.32^Bb^	266 ± 53.11^Bc^	305 ± 63.22^Ba^
N4	245 ± 17.21^Ad^	299 ± 35.32^Ab^	274 ± 55.63^Ac^	310 ± 98.32^Aa^
DTH				
N1	125 ± 10.10^Bb^	121 ± 15.21^Cb^	115 ± 9.32^Cb^	125 ± 12.32^Ca^
N2	127 ± 10.21^Bb^	125 ± 21.21^Bb^	118 ± 30.21^Bc^	127 ± 12.55^Ca^
N3	127 ± 9.32^Bb^	132 ± 11.21^Ba^	118 ± 23.21^Bb^	131 ± 12.89^Ba^
N4	131 ± 9.66^Ab^	133 ± 10.21^Ab^	128 ± 22.11^Ab^	144 ± 16.32^Aa^
DTM				
N1	133 ± 12.22^Cc^	132 ± 22.32^Bc^	136 ± 25.52^Bb^	145 ± 12.32^Ba^
N2	132 ± 16.99^Cb^	133 ± 28.96^Bb^	135 ± 23.63^Bb^	148 ± 15.32^Ba^
N3	136 ± 18.98^Bc^	134 ± 29.66^Bc^	138 ± 22.32^Bb^	149 ± 15.63^Ba^
N4	140 ± 16.65^Ac^	144 ± 35.35^Ab^	145 ± 18.99^Ab^	152 ± 18.98^Aa^

Means provided with error bars. The small letter indicates a significant (*p* ≤ 0.05) difference between Zn treatments and capital letters for N_2_.

For an abbreviation description see the list at the start of the manuscript.

### Photosynthetic pigments

The photosynthetic pigment Chl *a* was notably increased as the levels of N and Zn were increased. However, the most apparent change was evaluated due to N3:T3 and N4:T4. The Chl *b* contents were more under moderate supplementation of N1 and Zn (T2, T3), while no change occurred at N4:T4. The Chl *b* pointedly decreased as levels of Zn increased under N2 supplementations, while N3:T2 showed the least amount of the Chl *b* and N4 applications represented no substantial changes. The amount of chlorophyll a/b was maintained at N1 and various Zn fertigation, while other levels (N3, N4) were considerably increased as the level of Zn increased. The total chlorophyll had shown maximum mean values at N3 and N4 with a combined application of Zn. The carotenoid pigments were decreased in response to elevated levels of Zn and N, except in N1: Zn.

### Shoot ionic contents

**Figure 1:**
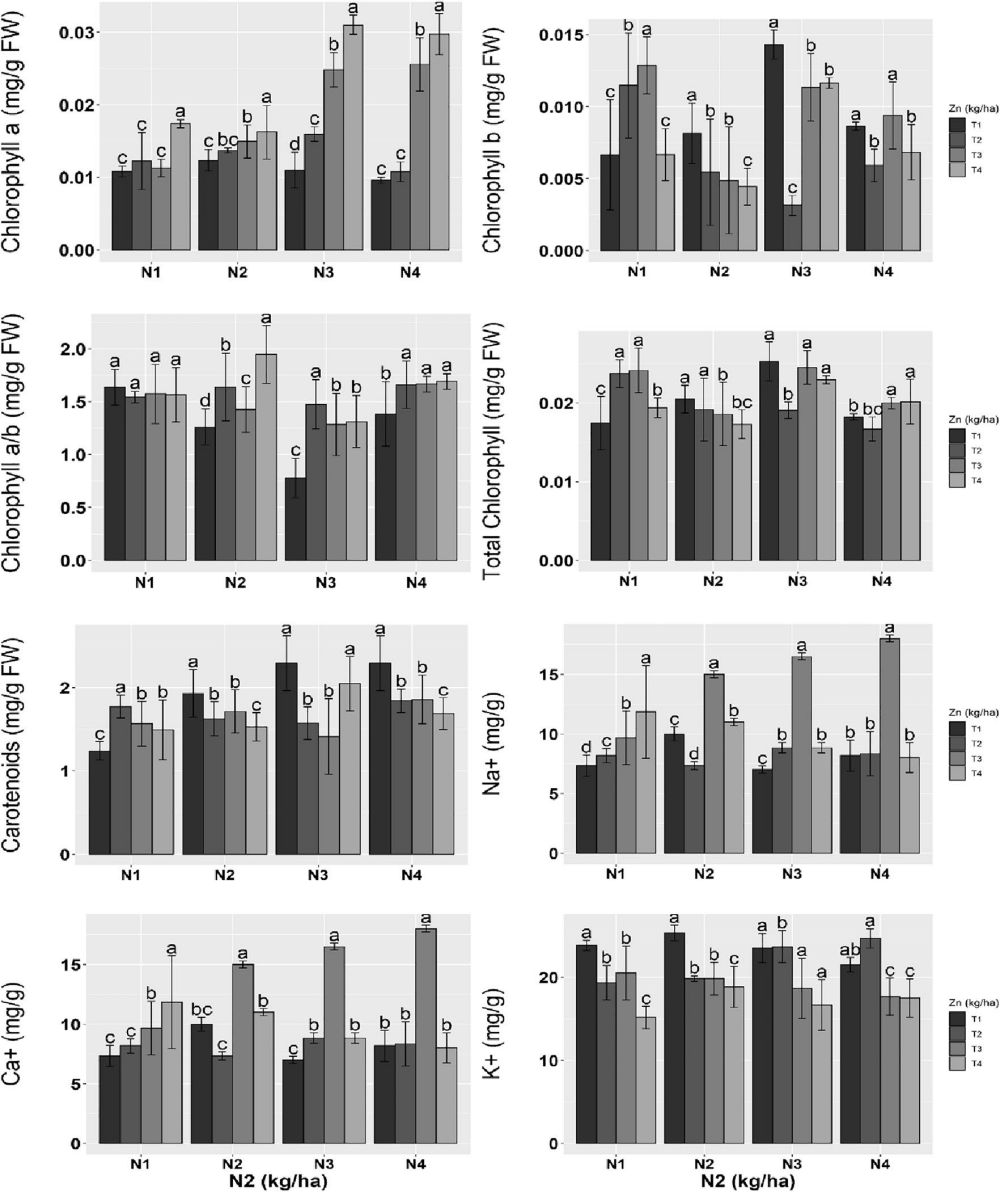
Photosynthetic pigments and shoot ionic contents of wheat treated with different levels of Zn and N2. Means ± SE provided with error bars; different letter indicates significance (*p* ≤ 0.05) between N and Zn treatments. For an abbreviation description see the list at the start of the manuscript.

The Na^+^ in the shoot increased in response to combined treatments; N4:T1, N4:T2, and N4:T4 maintain the Na^+^ contents. The Ca^2+^ was notably increased at the T3 level of Zn with combined fertigation of N. The N applications maintained the amount of K^+^ in shoots; however, a substantial reduction occurred at elevated levels of N and Zn (Fig. 1).

### Oxidative markers and antioxidants

The malondialdehyde contents in terms of lipid peroxidation were considerably reduced at higher applications of N and Zn to maintain the membrane integrity. The N3 and N1 applications with different levels of Zn substantially reduced reactive oxygen species (H_2_O_2_) levels, while higher levels of N and Zn showed an increasing pattern of reactive oxygen species.

**Figure 2:**
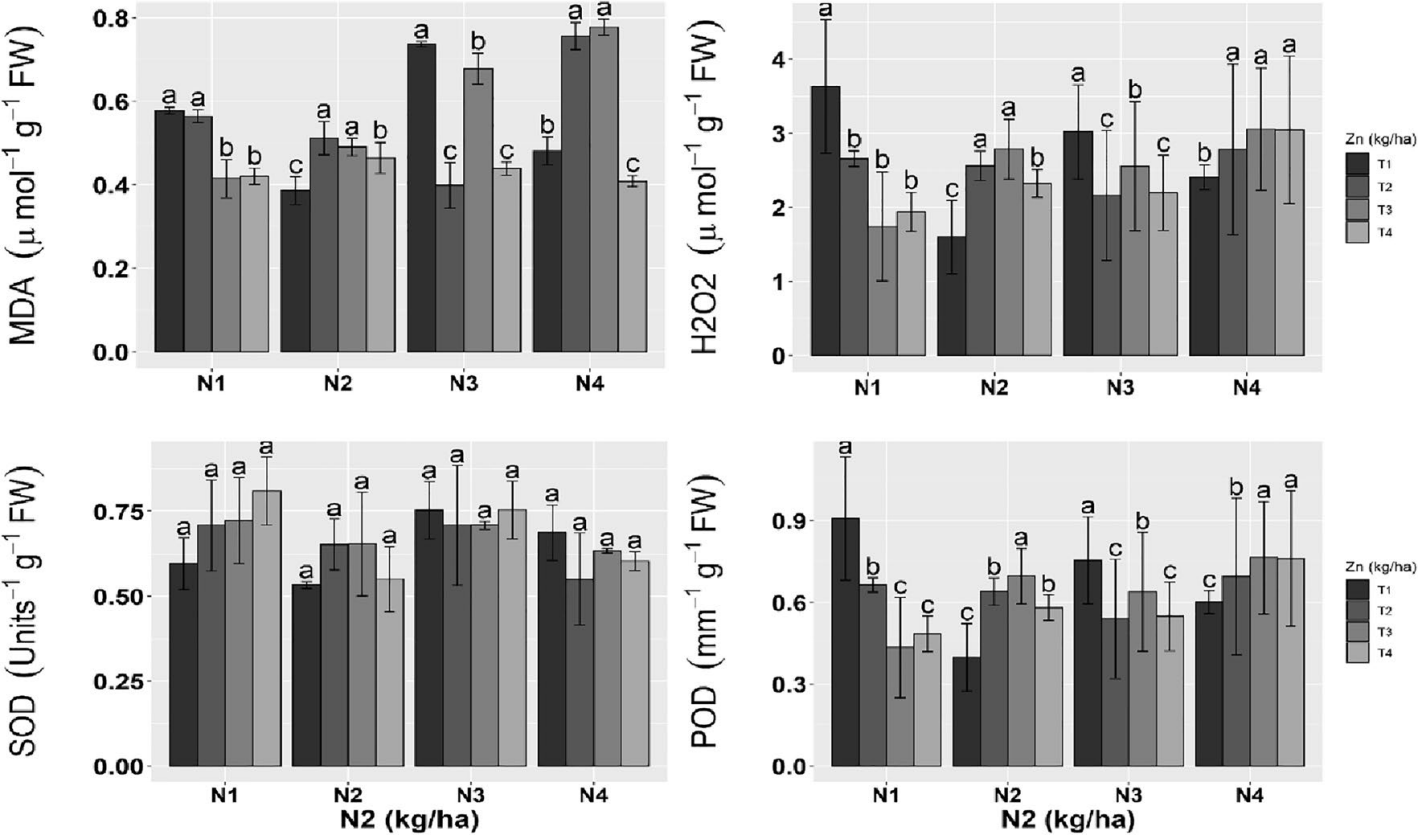
Oxidative stress markers (MDA, H_2_O_2_) and cellular antioxidants of wheat treated with different levels of Zn and N2. Means ± SE provided with error bars; different letter indicates significance (*p* ≤ 0.05) between N and Zn treatments. For an abbreviation description see the list at the start of the manuscript.

The cellular antioxidant^48^ was increased due to N1-N3 with different Zn levels. The amount of peroxidase considerably enhanced N2 and N4 applications with varying levels of Zn. A substantial reduction was evaluated in response to N1 and N2 levels with various Zn regimes (Fig. 2).

### Multivariate analysis

#### Principal component analysis (PCA)

The effect of various N and Zn combined applications on growth, pigments, ions, cellular antioxidants and ROS were illustrated with the help of ellipsed *PCAs.* The closed and linear eigenvector demonstrated a noteworthy association between traits at discriminating levels of N and Zn. The explained variation for growth and photosynthetic pigments were *PCA1* 57.3% and *PCA2* 11.2 with an influential cumulative of 68.5%. The photosynthetic pigments Chl *a,* Caro*, and* Chl *a/b* were closely clustered toward the PCA1 by closely associating with SFW, SPL, RFW and RL growth traits. These pigments were lowered with negative eigenvalues by associating with SL, NOS, Chl *a,* and DTM.

**Figure 3:**
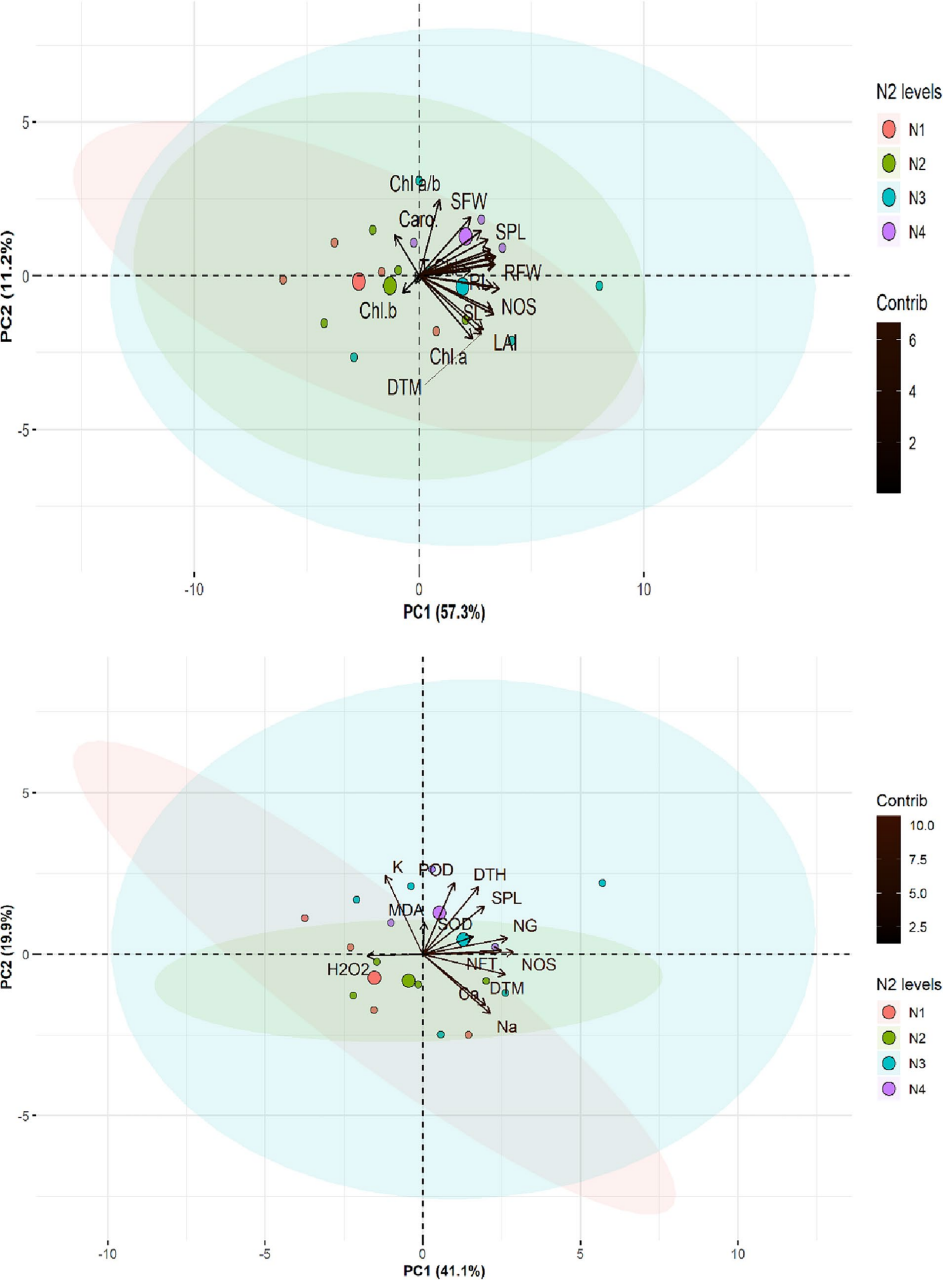
PCA biplot of a) growth, yield, and photosynthetic pigments traits b) shoot ions, antioxidants, ROS with yield traits of *Wheat* treated with different N_2_ and Zn levels (N1-0, N2-60, N3-120, and N4-180 kg ha^-1^). For an abbreviation description see the list at the start of the manuscript.

The PCA biplot for ions, antioxidants ROS, and yield components of wheat represented explained variations of PC1 41.1% and PC2 19.9% (Total 61%). The K^+^ and malondialdehyde (MDA) contents were closer by showing higher positive eigenvalues and excelled toward the PC2 side of the biplot. The traits POD, DTH, SPL, NG, and SOD were positively associated with each other and grouped toward the PC1 side of the biplot. The NOS, NFT, Ca^2+^ and Na^+^ were lowered, contributing to the negative side of PC1 with higher negative eigenvalues. The ROS was considerably associated with the various levels of the N by excelling toward the PC2 side (Fig. 3).

#### Pearson’s correlations and Clustered heatmap

**Figure 4:**
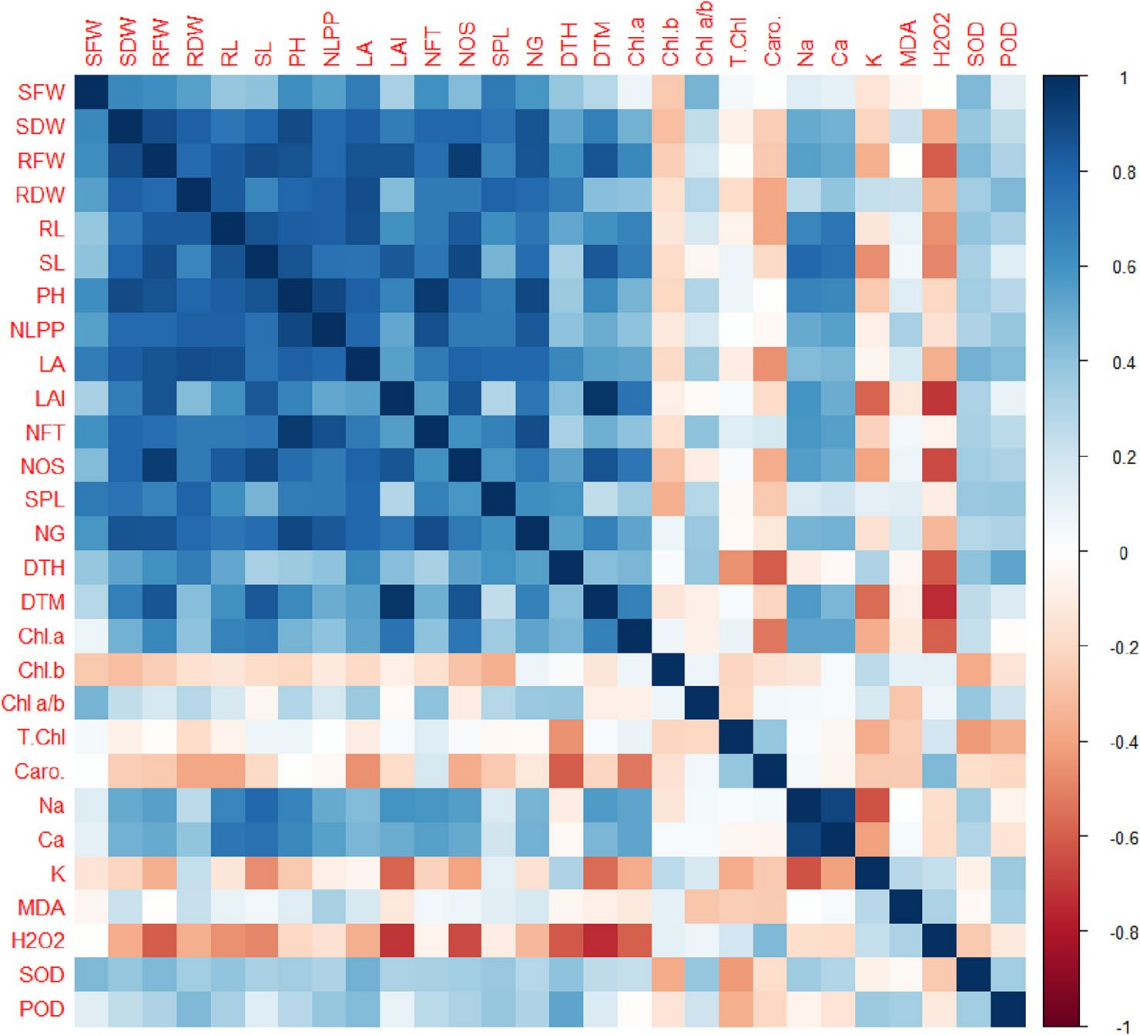
Pearson correlation matrix for growth, yield, photosynthetic pigments, shoot ions, antioxidants, and ROS traits of *Wheat* treated with different N_2_ and Zn levels. For an abbreviation description see the list at the start of the manuscript.

The Pearson correlation matrix revealed a considerable correlation among studied traits. The wheat growth traits as SFW, SDW, RFW, RDW, RL, SL, PH, NLPP, and LA were positively correlated with the yield components (NFT, NOS, SPL, NG, DTH, DTM), photosynthetic pigments (Chl *a,* Caro*,* T.Chl*),* antioxidant (SOD and POD) and shoot Ca^2+^. However, the growth was negatively associated with the MDA, ROS, and K^+^ (Fig. 4).

**Figure 5:**
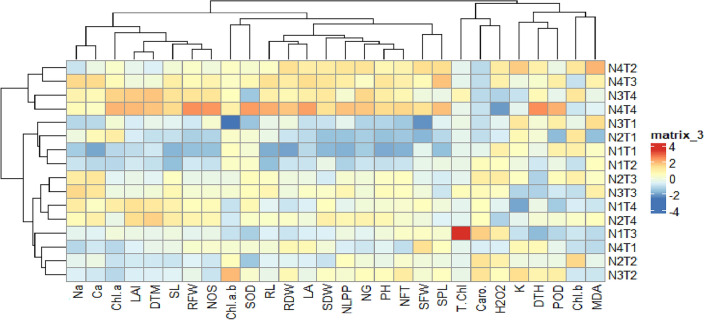
Clustered heatmap representing the influence of N_2_ and Zn applications on growth, yield, photosynthetic pigments, shoot ions, antioxidants, and ROS traits of *Wheat* treated with different N_2_ and Zn levels. N1-0, N2-60, N3-120 and N4-180 kg ha^−1^; T1-0, T2-10, T3-15 and T4-20 kg ha^-1^. For an abbreviation description see the list at the start of the manuscript.

A clustered heatmap was constructed to undertake the response of individual traits to N and Zn treatment. The Na^+^, Ca^2+^, Chl *a,* LAI, DTM, SL, RFW, NOS, Chl *a/b,* SOD, RL, RDW, LA, SDW, NLPP, NG, PH, NFT, SFW, and SPL were linked with N4T2, N4, T3, N3T4, N4T4, N2T3, N3T3, and N2T4, while these traits negatively responded to N3T1, N2T1, N1T2. The K^+^, DTH, POD, *Chl b, and* MDA were positively linked with N4T2, N4T3, N1T1, N1T2, and N2T3, while negatively interacted with N1T4, N1T3. All of the studied traits were closely clustered with each other in response to various N and Zn treatments (Fig. 5).

## Discussion

The present study evaluates the combined effect of N and Zn on crop growth, yield, pigments, ions, ROS, and antioxidants in wheat plants. For years, the synergetic role of the available nutrients to plants has been well established^49^. The micro (Zn) and macronutrient (N) deficit profile soil is increasing enormously due to exhaustive crops and injudicious use of fertilizers, leading to malnutrition in humans^50^. As a critical nutrient, the N plays a key role in Zn sequestrations in the wheat's vegetative and reproductive parts of the wheat^51,52^. Nitrogen as a macronutrient enhances vegetative growth by regulating the morphologic, physiological, and biochemical mechanisms^53^. The Zn plays an indispensable role in development by regulating the levels of antioxidants and nucleic acid metabolism, a key part of carbonic anhydrase enzymes involved in auxin biosynthesis^54,55^. In the current work, the synergistic application of Zn and N substantially improves plant biomass as shoot and root fresh (FW) and dry (DW) weights and plant height (PH) in wheat. The increase in fresh and dry biomass and height might occur due to elevated pigment levels and antioxidants. The increase in plant biomass is directly linked to the photosynthetic activity of the plants as N is a major component of the chlorophyll. The Zn essentially involves the biosynthesis of the proteins, the main part of ribosomes for their structural integrity and interaction with other elements involved in the growth of the plants. The N and Zn fertigation enhances wheat root length (RL) and shoot length. The increase in RL occurs to capture the immobilized Zn and N^56,57^. The SL increases the capacity to move nutrients toward aerial parts of the plant^58,59^. The increase in RL and SL mainly occurs due to the involvement of the Zn in cell division, cell proliferation and enhances the meristematic activities to facilitate the better translocation of nutrients across the root and shoot. Leaf area and numbers increase as the levels of Zn and N increase, this increase might occur to provide the maximum surface area to capture the solar light to enhance the photosynthesis. The exogenous supply of Zn and N increases the number of leaves and area in wheat^36^. The leaf area ratio (LAR) increased with increasing regimes of nutrients. The N and Zn applications in wheat markedly enhance the biomass by an increase in leaf area index, LAR, which ultimately helps the plant to increase the number of spikes and yield^60,61^.

Plant yield is closely correlated with key yield components such as numbers of fertile tillers (NFT) and spikes (NOS)^36^. Various studies demonstrated the positive role of the sole application of N and Zn in increasing the NOS and NFT^62–64^. In the present work, the NFT and NOS spikes increased, contributing to an increase in yield performance and a positive effect on grain numbers and quality. An increase in NFT and NOS is an important facet in determining the final yield per plant^65,66^. The spike length (SPL) increases in wheat plants. This increase is related to the greater number of grains per spike and the possible role of Zn in cell division, enlargement, and elongation^67^. In current work, the numbers of grains per spike have increased due to combined applications. This increase is linked to more NOS, NFT, and an increase in LAR. Improved supply of N and Zn work parallel to an increase in spike numbers, eventually leading to more seed numbers^68^. The grain yield confers a positive relation with days to heading (DTH) and maturity (DTM) in wheat^69^. The Zn as a microelement has an essential constructive and catalytic role in various physiological and biochemical activities which ultimately results in a higher economic yield in wheat^69^.

Enhanced amounts of photosynthetic pigments play a pivotal role in photosynthetic performance by capturing more light photons and providing energy to thylakoid membranes^70^. The amount of Chl *a*, *b*, Chl *a/b*, and total chlorophyll enhanced due to wheat's combined fertigation of N and Zn. The increasing trend of photosynthetic pigments improves photosynthetic efficacy in cereal crops, increases photochemical activity, and improves parabolic relations with an increase in yield^71^. The carotenoid in the leaves of wheat increases as the levels of N and Zn increase. The carotenoids in higher plants correlated with the supply of N and Zn to the plants, ultimately linked to the yield^71^. Carotenoids are a vital pigment, light absorption, distribution, and transmission to the reaction centre and play an important role in growth and development^72^. Ion homeostasis is a central plant quench that regulates the normal metabolic process^73^. The amount of Na^+^ in wheat under combined applications of N and Zn increased due to low supply while remaining constant at higher applications. The regulation of Na^+^ is undertaken by various mechanisms such as more Zn sequestration, avoidance of absorbing Na^+^, compartmentalization in vacuoles, and exploitation of osmotic adjustment approaches. The Zn regulate the balance of K^+^/Na^+^ by more accumulation of the K^+^ in pant vegetative parts to maintain internal ion homeostasis. Also, Zn enhances the adenosine triphosphate by regulating the Na^+^/H^+^ antiports for compartmentalization^74^. The N supplementation also plays a vital role in reducing the cellular Na^+^ and improving the K^+^ uptake in wheat^75^. The same pattern of increase in K^+^ concentration was recorded in the current work, which might be related to the controlling mechanism of Na^+^ due to N and Zn fertigation. As a divalent macromolecule, calcium plays an imperative role in membrane integrity, signaling, and sustaining the metabolism of crop plants^76^. The current illustration indicates a higher level of Ca^2+^, which is vital for normal metabolic processes and growth.

The Zn and N applications are dominant in scavenging reactive oxygen species (ROS) and free radicals and protecting membranes through lipid peroxidation by enhancing the amount of cellular antioxidants^77,78^. The antioxidants potentially reduce the production of the ROS cause a marked decrease in the amount of singlet oxygen and also elevate the organic osmolytes in cells. The activities of SOD enhanced as a result of N and Zn applications. This increase is interlinked with reducing LPX and H_2_O_2_ contents in the leaves. The increasing signature of SOD is supported by ROS limitation, which helps the plant counteract oxidative damages under Zn applications^79^. The rising capability of SOD is indispensable for the distribution of toxic radicles, protecting the membranes, photosynthetic apparatus, and nucleic acids, and providing functional stability as a result of N supplementation^75^.

## Conclusion

Fertigation of nitrogen (N) and zinc (Zn) notably increases wheat growth by enhancing the plant's fresh and dry biomass. Both nutrients also improve vegetative growth traits such as RL, SL, PH, NLLP, and ultimately lead to an increase in yield components. The increase in growth traits and yield components was closely linked with higher levels of photosynthetic pigments, assisted by N and Zn combined applications. Additionally, N and Zn tempt changes in the level of antioxidants, which perform a decisive role in scavenging the ROS as H_2_O_2_ and consequent decrease in lipid peroxidation to protect cellular membranes. The sole and combined applications of N and Zn efficiently maintain ionic statutes in shoots by balancing the Na^+^, Ca^2+^, and K^+^. The findings reported in this manuscript shed light on the intricate cross-talk between Zn and N, and unlock the pathways for future research to unravel molecular mechanisms. Moreover, further explorations by large-scale field experimentations could provide valuable insights into harnessing the role of N and Zn in agriculture.

## Data Availability

The original data are presented in this paper and do not include any supporting/ supplementary material. Any further queries can be directed to the corresponding author.

## Abbreviations

SFW: Shoot fresh weight
SDW: Shoot dry weight
RFW: Shoot fresh weight
RDW: Shoot dry weight
LA: Leaf area
RL: Root length
NLPP: Leaf per plant
LAR: Leaf area ratio
NFT: Number of fertile tillers
NOS: Numbers of spikelet’s
SPL: Spike length
NG: Numbers of grains
DTH: Days to heading
DTM: Days to maturity
Chl.*a*: Chlorophyll *a*
Chl.*b*: Chlorophyll *b*
T: Chl: Total Chlorophyll
Chl a/b: Chlorophyll a/b
Caro: Carotenoids
Na^+^: Sodium ions
Ca^2+^: Calcium ions
K^+^: Potassium ions
MDA: Malonaldehyde
SOD: Superoxide dismutase
POD: Per oxidase
